# MicroRNAs Modulate Interactions between Stress and Risk for Cocaine Addiction

**DOI:** 10.3389/fncel.2016.00125

**Published:** 2016-05-26

**Authors:** Menahem B. Doura, Ellen M. Unterwald

**Affiliations:** Center for Substance Abuse Research, Lewis Katz School of Medicine at Temple UniversityPhiladelphia, PA, USA

**Keywords:** microRNA, stress, cocaine, extended amygdala, corticotropin-releasing factor, brain-derived neurotrophic factor

## Abstract

Exposure to stress increases vulnerability to drug abuse, as well as relapse liability in addicted individuals. Chronic drug use alters stress response in a manner that increases drug seeking behaviors and relapse. Drug exposure and withdrawal have been shown to alter stress responses, and corticosteroid mediators of stress have been shown to impact addiction-related brain function and drug-seeking behavior. Despite the documented interplay between stress and substance abuse, the mechanisms by which stress exposure and drug seeking interact remain largely unknown. Recent studies indicate that microRNAs (miRNA) play a significant role in stress modulation as well as addiction-related processes including neurogenesis, synapse development, plasticity, drug acquisition, withdrawal and relapse. MiRNAs are short non-coding RNAs that function as bidirectional epigenetic modulators of gene expression through imperfect sequence targeted degradation and/or translational repression of mRNAs. They serve as dynamic regulators of CNS physiology and pathophysiology, and facilitate rapid and long-lasting changes to complex systems and behaviors. MiRNAs function in glucocorticoid signaling and the mesolimbic dopamine reward system, as well as mood disorders related to drug withdrawal. The literature suggests miRNAs play a pivotal role in the interaction between exposures to stress, addiction-related processes, and negative affective states resulting from extended drug withdrawal. This manuscript reviews recent evidence for the role of miRNAs in the modulation of stress and cocaine responses, and discusses potential mediation of the interaction of these systems by miRNAs. Uncovering the mechanism behind the association of stress and drug taking has the potential to impact the treatment of drug abuse and prevention of relapse. Further comprehension of these complex interactions may provide promising new targets for the treatment of drug addiction.

## Neurocircuits and Pathways Common to Stress and Cocaine Abuse

Considerable evidence supports the overlap between the stress and reward systems of the brain and that alterations in stress systems may contribute to increased liability to abuse drugs including cocaine (de Jong and de Kloet, 2004). Both repeated stress and psychostimulant dependence are associated with alterations in the mesocorticolimbic dopamine system, the medial prefrontal cortex glutamatergic corticolimbic circuit, and corticotropin-releasing factor (CRF) signaling in the ventral tegmental area (VTA; Piazza and Le Moal, 1997; Everitt and Wolf, 2002).

The transition from drug-taking behavior to addiction is characterized by a shift from positive drug reinforcement involving dopamine signaling to negative reinforcement involving the stress systems of the brain where drug-taking now removes the dysphoria, anxiety and negative emotional state experienced during abstinence/withdrawal (Koob and Le Moal, 2001). The extended amygdala, comprised of the bed nucleus of the stria terminalis (BNST), central nucleus of the amygdala and the nucleus accumbens shell, serves as a common circuit between drug reward and the negative emotional state experienced during abstinence/withdrawal (Alheid et al., 1998; Koob and Le Moal, 2001). The extended amygdala receives afferent connections form limbic brain structures including the basolateral amygdala and hypothalamus, and sends efferent projections to the medial ventral pallidum and the lateral hypothalamus (Heimer et al., 1991). The extended amygdala also has interconnections with the VTA and ventral striatum. The BNST contains a large number of dopamine and norepinephrine terminals, CRF terminals and cell bodies, neuropeptide Y terminals, and receives afferent connections from the prefrontal cortex (Allen et al., 1984; Phelix and Paull, 1990; Pacak et al., 1995; Kozicz, 2001; Koob, 2003).

The extended amygdala functions in both the positive reinforcing effects of drugs of abuse and the negative reinforcing effects of drug abstinence and withdrawal. Drugs of abuse including cocaine increase extracellular levels of dopamine in the nucleus accumbens shell (Pontieri et al., 1995). Further, withdrawal from cocaine increases extracellular concentrations of CRF in the extended amygdala (Koob et al., 1994). Rats receiving repeated injections of corticosterone acquire cocaine self-administration at lower cocaine doses relative to rats receiving vehicle (Deroche et al., 1997), and blockade of glucocorticoid and CRF receptors suppresses cocaine self-administration in rats (Piazza and Le Moal, 1996; Goeders, 1997). Taken together, these findings suggest considerable overlap between and cross-regulation of the stress and reward systems of the brain. Interaction between the stress and reward systems can contribute to responding to drugs of abuse and abstinence/withdrawal following exposure to drugs of abuse. Long-lasting changes in the stress and reward systems of the brain are known to play a crucial role in the transition from recreational drug taking to compulsive drug abuse. Understanding of the role of miRNAs in the maintenance of homeostasis within and between these brain systems will significantly improve our understanding of the etiology of compulsive drug use and further provide new genetic targets for the treatment of substance abuse.

## Micrornas Modulate Cocaine Reward and Withdrawal

Several recent studies demonstrate that miRNAs play a direct and crucial role in the modulation of cocaine intake in rodent models (Table 1). MiRNAs exert their regulatory translational repression and degradation of mRNA through the RNA-induced silencing complex (RISC). A core component of the RISC complex is the Argonaute (Ago) miRNA binding proteins, particularly Ago2 which mediates miRNA-dependent degradation and translational repression of target mRNAs (Hammond et al., 2001; Liu et al., 2004; Song et al., 2004) and functions in the generation of selective miRNAs from their precursors (Diederichs and Haber, 2007; O’Carroll et al., 2007). Mice deficient in Ago2 expression in dopamine D2 (Drd2) expressing neurons in the striatum self-administer significantly fewer infusions of cocaine with a downward shift in the cocaine dose-response curve relative to control animals expressing normal levels of Ago2 (Schaefer et al., 2010). Further, in contrast to wild-type control mice, Ago2-deficient mice show no preference for cocaine paired environments in conditioned place preference experiments (Schaefer et al., 2010). Deficient expression of Ago2 in the striatum results in decreased expression of ~25% of the examined miRNA species, providing strong evidence for the modulation of cocaine self-administration and reward by miRNAs through the action of Ago2 in the RISC complex. Thus, Ago2 plays a critical role in cocaine reward and motivation to self-administer cocaine. Further, these studies demonstrate that miRNAs function to modulate complex behavioral responses through region specific post-transcriptional regulation of gene expression.

**Table 1:** **Behavioral effects and molecular targets/regulators of microRNA(miRNAs) expression**.

miR	Expression effects	Targets/regulators
miR-181a	Overexpression increases cocaine CPP (Chandrasekar and Dreyer, 2011)	BDNF, SIRT1, GluA2 (Rivetti di Val Cervo et al., 2012; Saba et al., 2012)
miR-124/124a	Overexpression attenuates cocaine CPP (Chandrasekar and Dreyer, 2011)	Mineralocorticoid receptor, BDNF, CREB (Chandrasekar and Dreyer, 2009; Sõber et al., 2010)
miR-134	Modulates cocaine plasticity (Gao et al., 2010)	CREB, BDNF, SIRT1 (Gao et al., 2010)
miR-135a	Overexpression attenuates social defeat stress (Issler et al., 2014)	Mineralocorticoid receptor, serotonin transporter and receptor 1a (Sõber et al., 2010; Issler et al., 2014)
miR-375		BDNF, MAP3K8, POMC, CRF (Abdelmohsen et al., 2010; Zhang et al., 2013)
miR-212	Overexpression decreases cocaine self-administration Down-regulation increases cocaine self-administration (Hollander et al., 2010)	BDNF, CREB:TORC, MeCP2 (Hollander et al., 2010)
miR-183		mTor (Kye et al., 2014)
miR-9		BDNF, CREB, SIRT1 (Wu and Xie, 2006; Delaloy et al., 2010; Dajas-Bailador et al., 2012)
miR-26a/26b		BDNF, CREB (Caputo et al., 2011)
miR-449a	Overexpression decreases CRF-R1 expression Down-regulation attenuates CRF-R1 downregulation (Nemoto et al., 2012)	POMC, CRF-R1 (Nemoto et al., 2012)
miR-132		BDNF, CREB, glucocorticoids, ERK, SIRT1, glutamate receptor (Strum et al., 2009; Kawashima et al., 2010; Numakawa et al., 2011; Yi et al., 2014)
Let7	Modulates cocaine plasticity (Chandrasekar and Dreyer, 2009)	SIRT1 (Helwak et al., 2013)
miR-34c	Overexpression decreases anxiety-like behavior (Haramati et al., 2011)
miR-19b		Androgenic receptor B1, PTEN (Olive et al., 2009; Volk et al., 2014)

**Figure 1:**
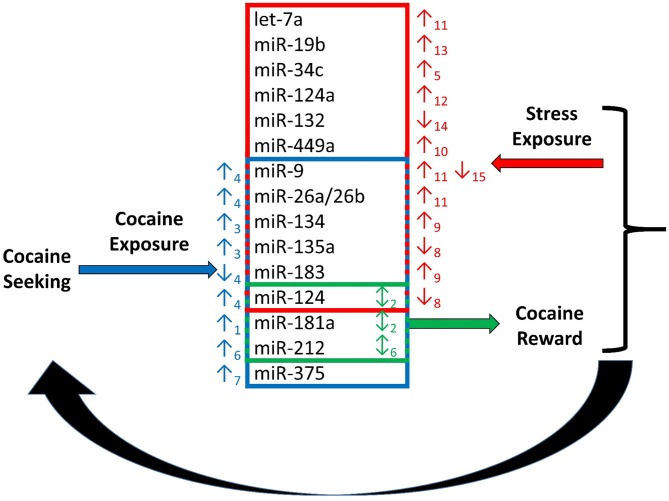
**Regulation of cocaine reward and stress by microRNAs (miRNAs).** Chronic cocaine exposure has been shown to alter expression of a number of miRNAs in various regions of the brain resulting in altered expression of down-stream molecular targets (Chandrasekar and Dreyer, 2009)_1_, (Chen et al., 2013)_3_, (Eipper-Mains et al., 2011)_4_, (Hollander et al., 2010)_6_, (Jonkman and Kenny, 2013)_7_. This in turn effects responding in the stress and reward systems of the brain. Exposure to stress regulates expression of miRNAs, and exogenous alteration of expression of several miRNAs regulated by cocaine and stress exposure alters the rewarding effects of cocaine. Alteration of miRNA expression in the stress and reward systems together modulate cocaine intake in a feed-back regulatory loop (Chandrasekar and Dreyer, 2011)_2_, (Haramati et al., 2011)_5_, (Hollander et al., 2010)_6_, (Mannironi et al., 2013)_8_, (Meerson et al., 2010)_9_, (Nemoto et al., 2012)_10_, (Rinaldi et al., 2010)_11_, (Shimizu et al., 2015)_12_, (Volk et al., 2014)_13_, (Yi et al., 2014)_14_, (Zhang et al., 2015)_15_.

**Figure 2:**
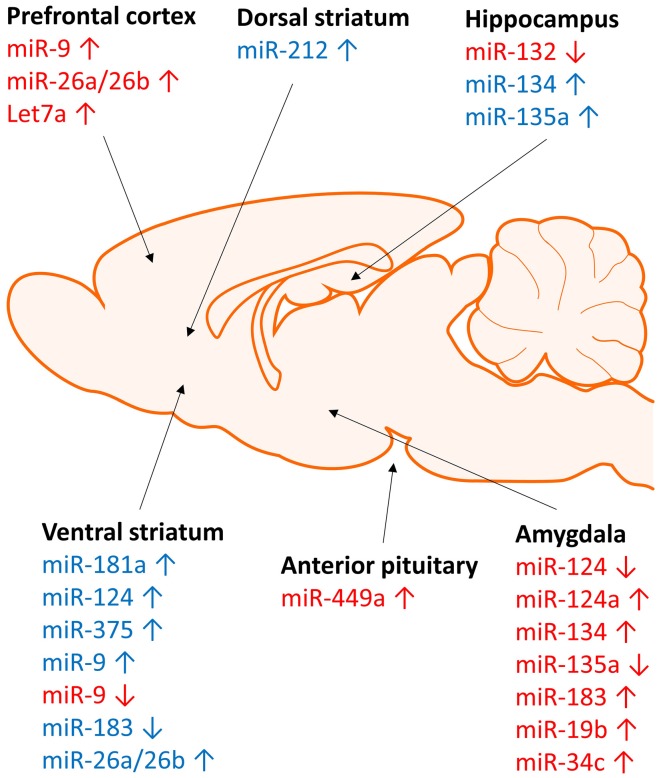
**Brain region specific regulation of micrRNA expression by stress and cocaine.** Regulation of microRNA expression by stress (red) and cocaine (blue) occurs in a region specific manner. Stress and cocaine exposure alters microRNA expression in brain regions involved in stress (amygdala), reward (striatum), and learning and memory (hippocampus and frontal cortex).

Exposure to cocaine significantly alters miRNA expression in many regions of the brain (Figures 1, 2). MiR-134 and miR-135a are upregulated in the hippocampus following exposure to cocaine (Chen et al., 2013). MiR-181a (Chandrasekar and Dreyer, 2009), miR-212 (Hollander et al., 2010), and miR-375 (Jonkman and Kenny, 2013) are upregulated in the striatum following cocaine exposure. MiR-9 and miR-124 are upregulated, whereas miR-183 is downregulated in striatal post-synaptic densities (Eipper-Mains et al., 2011). Further, altered expression of these miRNAs has been demonstrated to have profound effects on cocaine reward and intake (Table 1). For instance over-expression of miR-181a in the nucleus accumbens increases cocaine-induced conditioned place preference, whereas miR-181a knockdown has the opposite effect (Chandrasekar and Dreyer, 2011). Over-expression of miR-124 in the nucleus accumbens attenuates cocaine conditioned place preference (Chandrasekar and Dreyer, 2011). MiR-135a is upregulated 2.5-fold and miR-134 is upregulated greater than 7-fold following extinction of cocaine conditioned place preference (Chen et al., 2013). MiR-134 functions in memory formation and synaptic plasticity through sirtuin 1 (SIRT1), and regulates expression of cAMP response element binding protein (CREB) and brain-derived neurotrophic factor (BDNF) (Gao et al., 2010). Chronic cocaine induces SIRT1 expression in the nucleus accumbens (Ferguson et al., 2013). Over-expression of SIRT1 in the accumbens increases the rewarding effects of cocaine, and knockdown of SIRT1 has the inverse effect on cocaine reward (Ferguson et al., 2013). Further, a recent study by Quinn et al. (2015) identified several miRNAs predicted to function in synaptic plasticity which show differential expression in the dorsolateral and dorsomedial striatum of rats with high versus low propensity to self-administer cocaine. MiRs 101b, and 431 are significantly overexpressed, whereas miR-212 is significantly underexpressed in the dorsomedial striatum of the high cocaine responders. In the dorsolateral striatum, miRs 101b, 132, 181a, 431, and 708 are significantly overexpressed in vulnerable animals relative to those that show resilience to cocaine seeking (Quinn et al., 2015). These miRNAs may modulate responding to drugs of abuse through regulation of LTD, LTP, and specifically activity-regulated cytoskeleton-associated protein (Arc), a master regulator of synaptic plasticity.

Evidence shows that miR-212 plays a particularly important role in the modulation of cocaine intake. MiR-212 is upregulated 1.75-fold in the dorsal striatum of rats provided extended daily access to cocaine self-administration compared to restricted access and yoked rats (Hollander et al., 2010). Rats overexpressing miR-212 in the dorsal striatum have lower cocaine self-administration rates and a downward shift in cocaine self-administration dose response under extended access conditions relative to control animals, indicating a decreased motivation to self-administer cocaine. Inhibition of miR-212 increases cocaine intake and increases non-reinforced responding suggesting that miR-212 modulates cocaine intake and compulsive-like responding (Hollander et al., 2010). These actions are mediated by the regulation of CREB:TORC signaling by miR-212 in response to cocaine exposure (Hollander et al., 2010). The regulation of CREB signaling by cocaine is an established mechanism contributing to the modulation of the rewarding effects of cocaine (Carlezon et al., 1998; McClung and Nestler, 2003).

The kappa opioid system also plays a significant role in the modulation of cocaine reward. Dynorphin, an endogenous kappa opioid peptide, as well as exogenous kappa opioid agonists decrease dopaminergic tone in the striatum resulting in dysphoria and negative affective state (Tejeda et al., 2012). Chronic exposure to cocaine upregulates kappa opioid receptors and decrease dopaminergic activity (Unterwald et al., 1994; Maisonneuve et al., 1995). Further, stress induced activation of kappa opioid receptors through release of dynorphin potentiates the rewarding effects of cocaine as measured by conditioned place preference (McLaughlin et al., 2003). Thus far, there is little data in the literature on the regulation of kappa opioid receptors or prodynorphin by miRNAs. However, it is known that CREB regulates prodynorphin expression in rodents. Overexpression of CREB increases the expression of dynorphin whereas expression of mutant CREB has the opposite effect (Carlezon et al., 1998). Several of the miRNAs discussed herein regulate CREB, and likely function to regulate kappa opioid receptors and dynorphin through modulation of CREB activity.

In addition to CREB signaling, miRNAs also regulate BDNF which is known to function in the rewarding and reinforcing actions of cocaine. The effects of BDNF on cocaine reward and seeking behavior are brain region specific. BDNF infusion into the nucleus accumbens and VTA enhance the rewarding effects of cocaine (Lu et al., 2004; Graham et al., 2007). In contrast, BDNF infusion into the prefrontal cortex following cocaine self-administration attenuates cocaine reinstatement via modulation of ERK, CREB and glutamate signaling (Berglind et al., 2007, 2009; Whitfield et al., 2011). MiR-212 indirectly regulates BDNF levels in the striatum of mice undergoing extended access cocaine self-administration through interaction with MeCP2 (Im et al., 2010). MeCP2 expression is positively correlated with BDNF expression, and miR-212 expression is negatively correlated to BDNF expression (Im et al., 2010). Increased expression of CREB in the nucleus accumbens shell significantly increases cocaine self-administration and motivation to self-administer cocaine (Larson et al., 2011) and this may be related to BDNF regulation. Animals overexpressing CREB have increased expression of BDNF and a short-term upward and long-term leftward shift in the cocaine dose-response curve for IV self-administration. Further, increased CREB expression after withdrawal reinforced cocaine-stimulated reinstatement (Larson et al., 2011). Expression of BDNF is increased in midbrain and amygdala during withdrawal from cocaine self-administration, and is hypothesized to contribute to heightened motivation to self-administer cocaine (Grimm et al., 2003; Lu et al., 2004).

Increased BDNF activity in brain regions involved in drug reward potentiates the reinforcing effects of cocaine. BDNF infusions into the nucleus accumbens increases sensitivity to the psychomotor stimulant effects of cocaine (Horger et al., 1999) and increases cocaine self-administration behavior in rodents (Graham et al., 2007). Further, knockdown of BDNF in the accumbens decreases cocaine self-administration (Graham et al., 2009). Knockdown of MeCP2 results in increased expression of miR-212, whereas increased miR-212 expression decreased the expression of MeCP2 (Im et al., 2010). Striatal MeCP2 knockdown decreases cocaine self-administration and shifts the cocaine self-administration dose-response curve down in animals given extended access. Knockdown of miR-212 expression in the striatum of MeCP2 deficient mice restores cocaine self-administration and shifts the cocaine dose response curve back up to control levels (Im et al., 2010). This suggests miR-212 represses expression of MeCP2 and is itself repressed by MeCP2 thereby regulating cocaine effects on striatal BDNF expression. This negative homeostatic balance in turn regulates the rewarding properties of cocaine and may contribute to the escalation to compulsive drug seeking. Taken together, these data provide strong evidence that miRNAs serve as critical short-term and long-term epigenetic modulators of cocaine exposure and reward through regulation of canonical drug reward signaling cascades. Cocaine exposure alters miRNA expression in several brain regions, and modulation of regional expression of these miRNA species alters responding to cocaine in several behavioral paradigms of drug reward.

## Interactions of Cocaine-Seeking and Stress

In addition to the reward system, it is known that the stress systems of the brain play a vital role in drug seeking behavior. It is hypothesized that dysregulation of both the reward and stress pathways of the brain lead to the transition from recreational to compulsive drug seeking, and long-term dysregulation of these systems leads to vulnerability to relapse. Dopaminergic projections from the VTA to the medial prefrontal cortex have been implicated in the stress-induced relapse of cocaine-seeking in rodent reinstatement models (Vranjkovic et al., 2014). Dopamine D1 receptor activation in the VTA increases the activity of glutamatergic pathways leading to the nucleus accumbens, thereby increasing cocaine seeking (McFarland et al., 2004). Foot-shock stress increases CRF release in the VTA which in turn increases glutamate release activating mesocorticolimbic dopamine neurons and inducing cocaine reinstatement in rats previously exposed to cocaine self-administration (Wang et al., 2005). The VTA functions in the modulation of reward as part of the dopamine reward pathway and also receives inputs from brain regions involved in the modulation of stress response, including the extended amygdala (Phillipson, 1979). Of particular interest is the BNST which functions in the integration of stress and the reward system. Inhibition of the central nucleus of the amygdala, ventral BNST, and nucleus accumbens shell via co-infusion of baclofen and muscimol prevents reinstatement of cocaine seeking by foot shock stress (McFarland et al., 2004). Further, the BNST is critical in swim-stress induced reinstatement of cocaine seeking through CREB signaling (Briand et al., 2010). These data provide strong evidence that the extended amygdala is a critical region for the integration of stress and reward signaling in the brain and plays an important role in stress-induced drug-seeking behaviors. Recent evidence shows that miRNA expression is significantly altered in regions of the extended amygdala in response to acute and chronic stress. Many of these miRNAs are also modulated by cocaine exposure in reward regions thus suggesting that miRNAs regulate both reward and stress signaling in the brain. These complex interconnected regulatory cascades likely contribute to the long-term dysregulation of the reward and stress systems hypothesized to drive compulsive drug seeking.

## Micrornas Modulate Stress Response and Negative Affective States

MiRNAs play a role in the short-term and long-term modulation of stress response and contribute to the etiology of anxiety and depression-like behaviors. Several miRNAs have been identified that function in the modulation of stress responses including miRNAs also shown to function in cocaine reward acting through similar signaling pathways to affect stress response as well as drug reward (Table 1). Mir-134 and miR-183 expression is increased in the central nucleus of the amygdala in response to acute immobilization stress, and miR-183 modulates expression of SC35, a protein which regulates stress-induced alternative splicing of acetylcholinesterase *in vitro* (Meerson et al., 2010). Mannironi et al. (2013) demonstrated that miR-135a and miR-124 are significantly down-regulated in mouse amygdala following 2 h restraint and directly regulate expression of the mineralocorticoid receptor, a regulator of early stress response. In addition, miR-124 is upregulated by cholinergic agonists, and plays a critical role in the cholinergic anti-inflammatory pathway (Sun et al., 2013). Further, miR-375, which is upregulated by repeated cocaine exposure, inhibits proopiomelanocortin (POMC) expression by targeting MAP3K8 and mediating CRF signaling (Zhang et al., 2013). Acute stress has also been shown to alter expression of let-7a, miR-9 and miR-26a/b in the frontal cortex (Rinaldi et al., 2010), and miR-124a (Shimizu et al., 2015) in mouse corpus callosum. Expression of these same miRNAs is also altered in the striatum by cocaine exposure (Eipper-Mains et al., 2011).

The literature shows that these miRNAs, which are modulated by both drug and stress exposure, in turn modulate drug seeking and stress response through transcriptional regulation of downstream targets in interacting canonical pathways (Table 1). For instance, chronic unpredictable stress decreases expression of miR-9 in the nucleus accumbens leading to increased expression of the dopamine D2 receptor (Zhang et al., 2015). Restraint stress significantly increases expression of miR-449a, increases expression of POMC mRNA, and decreases expression of CRF-R1 mRNA and protein in the anterior pituitary of rats (Nemoto et al., 2012). Further, over-expression of miR-449a results in suppression of CRF-R1 mRNA and protein, and down-regulation of miR-449a attenuates suppression of CRF-R1 by dexamethasone in cultured anterior pituitary cells (Nemoto et al., 2012). This suggests that miR-449a contributes to the stress-induced down-regulation of CRF-R1 by glucocorticoids in the anterior pituitary. MiR-132 is upregulated by BDNF (Numakawa et al., 2011) and down-regulated by glucocorticoids (Kawashima et al., 2010) in cultured cells. Yi et al. (2014) have shown that miR-132 is down-regulated in the hippocampus of mice exposed to chronic unpredictable mild stress. Further, evidence suggests that miR-132 expression is regulated by the ERK signaling pathway (Remenyi et al., 2010). MiR-132 also modulates Toll-like receptor (TLR) signaling pathways via regulation of acetylcholinesterase to increase acetylcholine-mediated negative regulation of TLR-signaling pathways (Shaked et al., 2009).

MiR-34c is upregulated in the amygdala of mice exposed to acute restraint stress and repeated social defeat stress, and over-expression of miR-34c in the amygdala protects against the anxiogenic effects of acute restraint stress possibly through regulation of CRF-R1 (Haramati et al., 2011). MiR-19b selectively associates with Ago2, is significantly upregulated in the amygdala of mice exposed to chronic social defeat stress, and regulates expression of adrenergic receptor β1 *in vitro* (Volk et al., 2014). MiR-124a is upregulated in the hippocampus of adult rats exposed to social defeat stress (Bahi et al., 2014). MiR-124a is a direct regulator of BDNF expression, and as expected, BDNF is down-regulated in hippocampus of rats after social defeat stress, and over-expression of miR-124a in rat hippocampus exacerbates social defeat stress-induced depression-like behaviors as measured by novelty suppressed feeding, sucrose preference and force swim tests (Bahi et al., 2014). Recently, it was demonstrated that both activation of VTA- accumbens neurons and subthreshold social defeat stress stimulation are required to induce upregulation of BDNF in a CRF receptor dependent manner in the nucleus accumbens (Walsh et al., 2014). Further, accumbens BDNF promotes increases in cocaine self-administration and relapse (Graham et al., 2007). Dwivedi et al. (2015) have shown that chronic administration of corticosterone differentially regulates expression of 26 miRNAs in the prefrontal cortex, several of which (miRs 19b, 124, 181a, 135a) regulate responding to cocaine exposure. The majority of these miRNAs show binding sites for glucocorticoid receptor elements, suggesting a common regulatory pathway for miRNA regulation of corticosterone able to cross-talk with reward and synaptic plasticity pathways. MiR-181 suppresses TNF-induced cytokine production through regulation of p300/cyclic AMP response element binding protein-associated factor, demonstrating an integral role of miRNAs in inflammatory and immune responses (Zhao et al., 2012). These data suggest miRNAs shown to modulate cocaine-seeking behaviors, also contribute to the regulation of stress response, anxiety-like behaviors and anhedonia. Further this strongly suggests a role for miRNAs in the modulation of the observed interactions between stress exposure/response and drug reward.

## Summary

MicroRNAs (miRNAs) play significant roles in the modulation of both the stress and reward systems of the brain. The literature has long documented that both the reward and stress systems function in responding to the acute effects of drugs of abuse, as well as withdrawal/abstinence from chronic drug exposure. Chronic exposure to cocaine results in dysregulation of brain reward circuitry and recruitment of the brain stress systems leading to long-term cocaine dependence. Long lasting changes in both reward and stress systems lead to increased vulnerability to relapse during periods of abstinence. Several miRNAs have been identified which are co-regulated by chronic cocaine exposure and various models of rodent stress responding. These include, but are not limited to miR-134, 135a, 375, and the miR-212/132 family. Further, these miRNAs function in signaling pathways long known to regulate reward and stress response such as dopamine and CRF signaling, and glutamate transmission. It is well-established that CREB, CRF, and BDNF, among other molecules, play key roles in both stress and drug seeking/abuse. These molecules are therefore widely theorized to mediate the interaction between stress and drug seeking/abuse. Further, many of the miRNAs shown to regulate these molecules also function in immune and inflammatory responses, mechanisms known to play vital roles in the etiology of addiction. For instance, miR-132 and miR-212, shown to play a critical role in cocaine self-administration, also play important roles in TLR2 ligand-mediated TNF-α secretion (Nahid et al., 2013). The kappa opioid system is also known to modulate both stress response and the rewarding properties of drugs of abuse. However, to date, few studies have examined the regulation of kappa opioid signaling by miRNAs. These interactions are currently not very well understood, however, deciphering these complex signaling pathways will be vital to furthering our understanding of the addicted brain.

This review posits that miRNAs serve as master regulators of both the stress and reward systems through coordinated regulation of a large number key molecules and facilitate cross-talk between stress, reward, synaptic plasticity, and immune/inflammatory responses. Through integration of these signaling pathways, miRNAs serve as master regulators of downstream behavioral and cellular responses to drugs of abuse. In turn, miRNA expression is itself regulated by external stimuli including stress and drug exposure. MicroRNAs are highly conserved between species, however, species specific differences do exist. Within species, there are tissue and cell type differences in expression regulated by post-transcriptional modification of more ubiquitously expressed pre-miRNAs. This is likely necessary due to the ability of a single miRNA to regulate numerous target genes. While the complexity of miRNA regulation of gene expression poses a daunting challenge, it is an area of study that holds immense promise for future advancement of drug abuse research and the biomedical sciences as a whole. We argue that miRNAs co-regulated by stress and chronic cocaine represent prime targets for study in order to further elucidate the etiology of the transition from casual drug use to drug dependence. Further, this population of miRNAs represents promising targets for identification of novel treatments for drug abuse.

## Author Contributions

MBD and EMU wrote the manuscript.

## Conflict of Interest Statement

The authors declare that the research was conducted in the absence of any commercial or financial relationships that could be construed as a potential conflict of interest.

## Abbreviations

Ago: argonaute
Arc: activity-regulated cytoskeleton-associated protein
BDNF: brain derived neurotrophic factor
BNST: bed nucleus of the stria terminalis
CREB: cAMP response element-binding protein
CRF: corticotrophin releasing factor
Drd2: D2 dopamine receptors
ERK: Extracellular signal-regulated kinase
MAP3K8: mitogen-activated protein kinase kinase kinase 8
MeCP2: methyl CpG binding protein 2
POMC: proopiomelanocortin
RISC: RNA-induced silencing complex
SIRT: NAD-dependent deacetylase sirtuin
TLR: Toll-like receptor
TNF: tumor necrosis factor
TORC: target of rapamycin complex
VTA: ventral tegmental area.
